# Telephone interviews and online questionnaires can be used to improve neurodevelopmental follow-up rates

**DOI:** 10.1186/1756-0500-7-219

**Published:** 2014-04-08

**Authors:** Samantha Johnson, Sarah E Seaton, Bradley N Manktelow, Lucy K Smith, David Field, Elizabeth S Draper, Neil Marlow, Elaine M Boyle

**Affiliations:** 1 Department of Health Sciences, University of Leicester, 22-28 Princess Road West, Leicester LE1 6TP, UK; 2 Department of Academic Neonatology, Institute for Women’s Health, University College London, London, UK

**Keywords:** Neurodevelopmental outcomes, Follow-up, Response rates, Cohort study, Questionnaire

## Abstract

**Background:**

Maximising response rates to neurodevelopmental follow-up is a key challenge for paediatric researchers. We have investigated the use of telephone interviews and online questionnaires to improve response rates, reduce non-response bias, maintain data completeness and produce unbiased outcomes compared with postal questionnaires when assessing neurodevelopmental outcomes at 2 years.

**Methods:**

A prospective cohort study of babies born ≥32 weeks gestation. Neurodevelopmental outcomes were assessed at 2 years of age using a parent questionnaire completed via post, telephone or online. Relative Risks with 95% confidence intervals (RR; 95% CI) were calculated to identify participant characteristics associated with non-response and questionnaire response mode (postal vs. telephone/online). The proportion of missing data and prevalence of adverse outcomes was compared between response modes using generalized linear models.

**Results:**

Offering telephone/online questionnaires increased the study response rate from 55% to 60%. Telephone/online responders were more likely to be non-white (RR 1.6; [95% CI 1.1, 2.4]), non-English speaking (1.6; [1.0, 2.6]) or have a multiple birth (1.6; [1.1, 2.3]) than postal responders. There were no significant differences in the prevalence of adverse neurodevelopmental outcomes between those who responded via post vs. telephone/online (1.1; [0.9, 1.4]). Where parents attempted all questionnaire sections, there were no significant differences in the proportion of missing data between response modes.

**Conclusions:**

Where there is sufficient technology and resources, offering telephone interviews and online questionnaires can enhance response rates and improve sample representation to neurodevelopmental follow-up, whilst maintaining data completeness and unbiased outcomes.

## Background

Minimising attrition and maximising response rates present major challenges for researchers conducting longitudinal studies. This is particularly evident in the field of paediatrics, in which birth cohorts are frequently followed up for epidemiological studies and randomised trials of perinatal interventions. Participant attrition not only reduces the power of a study, but can bias results [1]: non-responders typically have greater medical and socio-economic risk and may differ systematically in terms of disorders of interest, resulting in biased estimates of adverse outcomes [2-7].

Postal questionnaires are frequently used to assess neurodevelopmental outcomes in large-scale studies and strategies for improving response rates have been well documented [8,9]. In particular, offering telephone interviews in addition to postal questionnaires has been shown to improve response rates in health related research [10,11]. Telephone interviews have also been shown to yield comparable or lower levels of missing data than postal questionnaires, thus improving the completeness of data obtained [11-14]. Offering telephone follow-up to non-responders to postal surveys has also been shown to improve response rates among participants from socio-economically deprived and ethnically diverse sectors of the population [15,16]. Therefore employing multiple modes of data collection may also improve sample representation and reduce non-response bias [10,11]. Electronic data collection methods are becoming increasingly popular and, like telephone interviews, these have also been shown to improve response rates over the use of postal questionnaires alone [13,17,18]. However, although mixed mode data collection may improve response rates, the mode of response may bias outcomes [10,13]. Thus is it important to explore the effects of response mode on both response rates and measures of data quality.

These issues have not been studied in relation to methods for improving response rates to infant neurodevelopmental follow-up. In particular, the impact of employing mixed mode data collection on parent-reported neurodevelopmental outcomes requires investigation as both parent and child characteristics may affect response rates and reported outcomes. Using parent reports of neurodevelopment at two years of age, we have explored the use of telephone interviews and online questionnaires for (1) increasing response rates, (2) improving sample representation, (3) maintaining data completeness and (4) yielding unbiased outcomes over postal questionnaires alone.

## Methods

### Population

The mothers of all babies born late and moderately preterm (32^+0^ to 36^+6^ weeks of gestation) from September 2009 to December 2010 and resident in a geographically-defined area of Leicestershire and Nottinghamshire were invited to participate in the Late And Moderately preterm Birth Study (LAMBS). A random sample of term-born babies (≥37 weeks gestation) was also recruited from the same region during the same time period. Mothers were approached as soon as possible after delivery to obtain informed consent and to carry out a maternal interview prior to their discharge from hospital. For mothers who were discharged in the first few hours after birth, a research midwife arranged a home visit as soon as possible after discharge in order to obtain consent. Contact was made as soon as possible in this way in order to minimise recall bias during the maternal interview. There were no exclusion criteria. Only data relating to live births are included in the present report. The study was approved by the Derbyshire NHS Research Ethics Committee (Ref 09/H0401/25).

### Procedure

**Figure 1:**
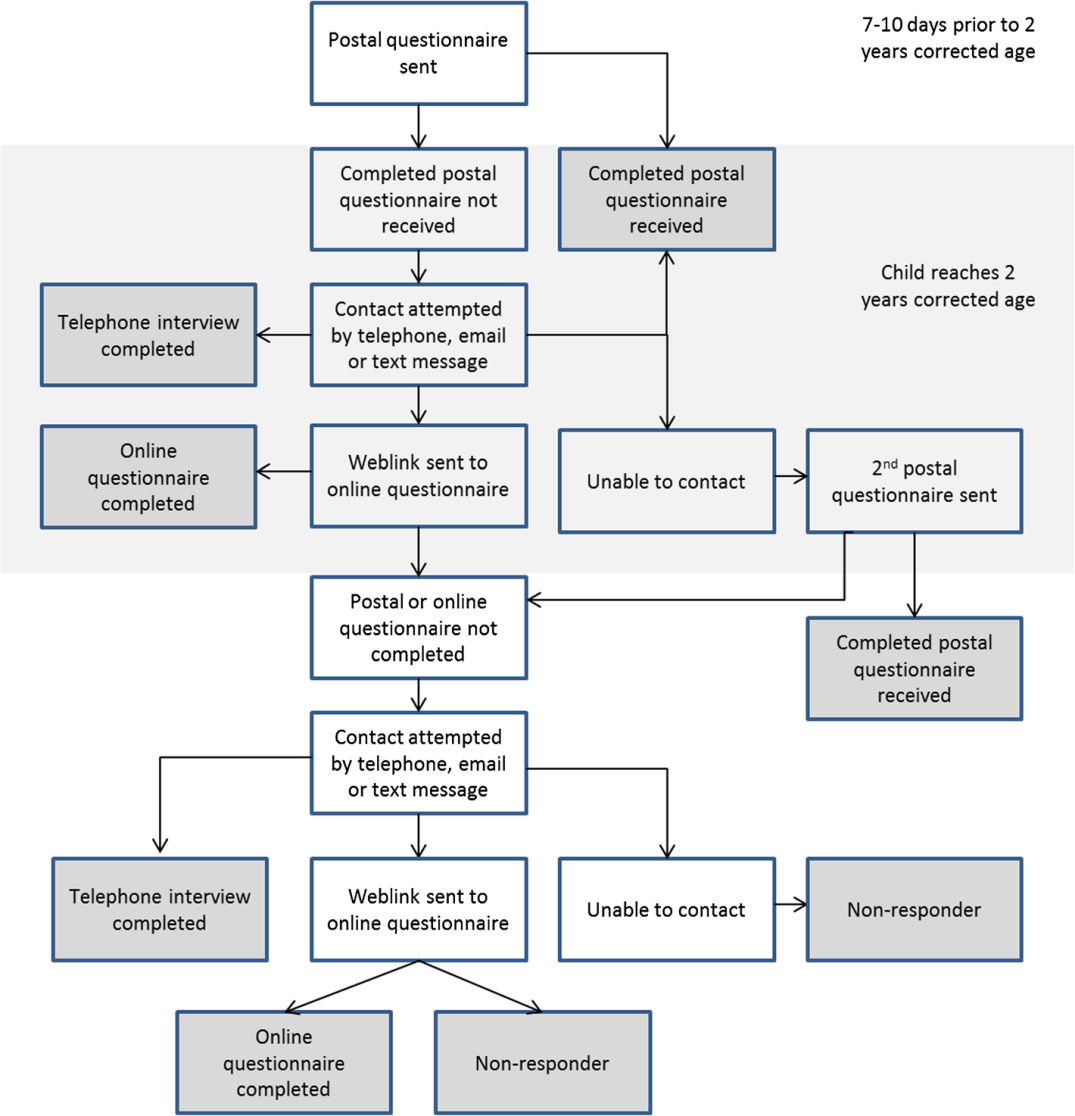
Procedure for obtaining 2-year follow-up data.

At the time of consent, mothers were interviewed to obtain information about socio-economic factors and antenatal health. Information about the baby’s hospital admission was collected from medical notes and parents were asked to complete a postal questionnaire to assess their child’s use of healthcare services at 6 and 12 months of age. The present report focuses on the 2-year neurodevelopmental follow-up for which data were collected using a hierarchical mixed-mode approach. Seven to ten days before the child turned 2 years of age corrected for prematurity (2 y-CA), parents were sent a postal questionnaire to complete. If no response was made by the time the child reached 2 y-CA, parents were then initially contacted by telephone to remind them that a questionnaire had been posted, to ask whether help was needed in completing it and to offer the option of telephone or online completion. If this contact could not be made by telephone after repeated unsuccessful attempts, an attempt was made to contact the parent by email or text message (where details were available) to provide this reminder. Where parents opted for a telephone interview, these were carried out at a time convenient to them; interviews were carried out in the evening where parents specified this was the most appropriate time. For families in which no member was able to speak English sufficiently to answer the questionnaire, a member of the study team who spoke the parents’ language carried out the interview where possible. Parents who requested online completion were emailed a web-link to an electronic version of the questionnaire hosted on surveymonkey.com [19]. If a completed questionnaire was not received 2 weeks later, parents were contacted again to prompt them to complete it and to again offer alternative modes of completion. At this point, parents who could not be contacted by telephone, text message or email were sent a second postal questionnaire. After a further 1-2 weeks, a final attempt was made to contact parents and offer alternatives for completing the postal questionnaire. Parents for whom a completed questionnaire was never received and those who could not be contacted despite repeated attempts were classified as non-responders (see Figure 1).

### Measures

Information obtained from the maternal interview was used to classify mothers’ occupation using the UK National Statistics Socio-Economic Classification (NS-SEC) [20] from which occupations were classified as managerial, professional/intermediate, routine/semi-routine or unemployed. Mothers’ highest educational qualification was classified as none or secondary level vs. tertiary qualifications. Mothers were also asked about their general health (categorised excellent/good/fair vs. poor), mental health (no anxiety/depression vs. moderately to severely anxious/depressed) and their financial situation (doing well/OK vs. finding it difficult) during pregnancy. Information collected from the infant’s medical notes included gestational age, birthweight, multiple birth status, fetal growth restriction (classified using customised antenatal growth charts [21]), congenital anomalies, respiratory support, feeding and cranial MRI and ultrasound findings. Intra-cranial abnormalities were classified where Grade III or IV intra-ventricular haemorrhage (IVH), periventricular leukomalacia (PVL) and Grade II or III neonatal encephalopathy were present.

Two-year questionnaires were identical for all modes of completion. The prevalence of adverse neurodevelopmental outcomes and the proportion of missing data were calculated for two outcome measures.

(1) Parent Report of Children’s Abilities-Revised (PARCA-R) [22], a 158-item parent report of cognitive and language development from which scores <49 [23] were used to identify cognitive delay. As blank items could be a valid response on the language sub-scale, the proportion of missing data was calculated for the 34-item non-verbal subscale alone.

(2) An additional four forced-choice items were used to assess the child’s communication, motor, vision and hearing function. Children with a moderate to severe impairment in any one of these domains were classified with neurosensory impairment (NSI) using standard criteria [24].

### Statistical analyses

Data were double entered then verified and analysed using Stata v12. Data obtained from mothers of late and moderately preterm and term-born infants were combined to analyse follow-up rates for the study as a whole. Risk Ratios (RR) for non-response, with 95% confidence intervals (95% CI), were estimated for maternal and infant characteristics; the same methods were used to estimate RRs for completing the questionnaire via telephone or online for the same characteristics. To assess the completeness of data obtained by response mode (postal vs. telephone/online), the percentage of missing data on each outcome measure was computed using a published formula [11]: number of missing items/[(number of responders) × (number of questions presented)]. The percentage of missing data with RR (95% CI) for absence of data were calculated using generalised linear models including robust variance estimation to account for clustering within questionnaires [25]. The percentage of children with adverse outcomes was calculated by response mode (postal vs. telephone/online) both unadjusted and after adjustment for characteristics associated with mode of response to determine whether mode of completion biased outcomes on each of the two measures. For all analyses a p-value <0.05 was taken as evidence of a statistically significant effect.

## Results

Results are presented in relation to the 4 study aims:

1) *Do telephone interviews and online questionnaires increase study response rates?*

**Figure 2:**
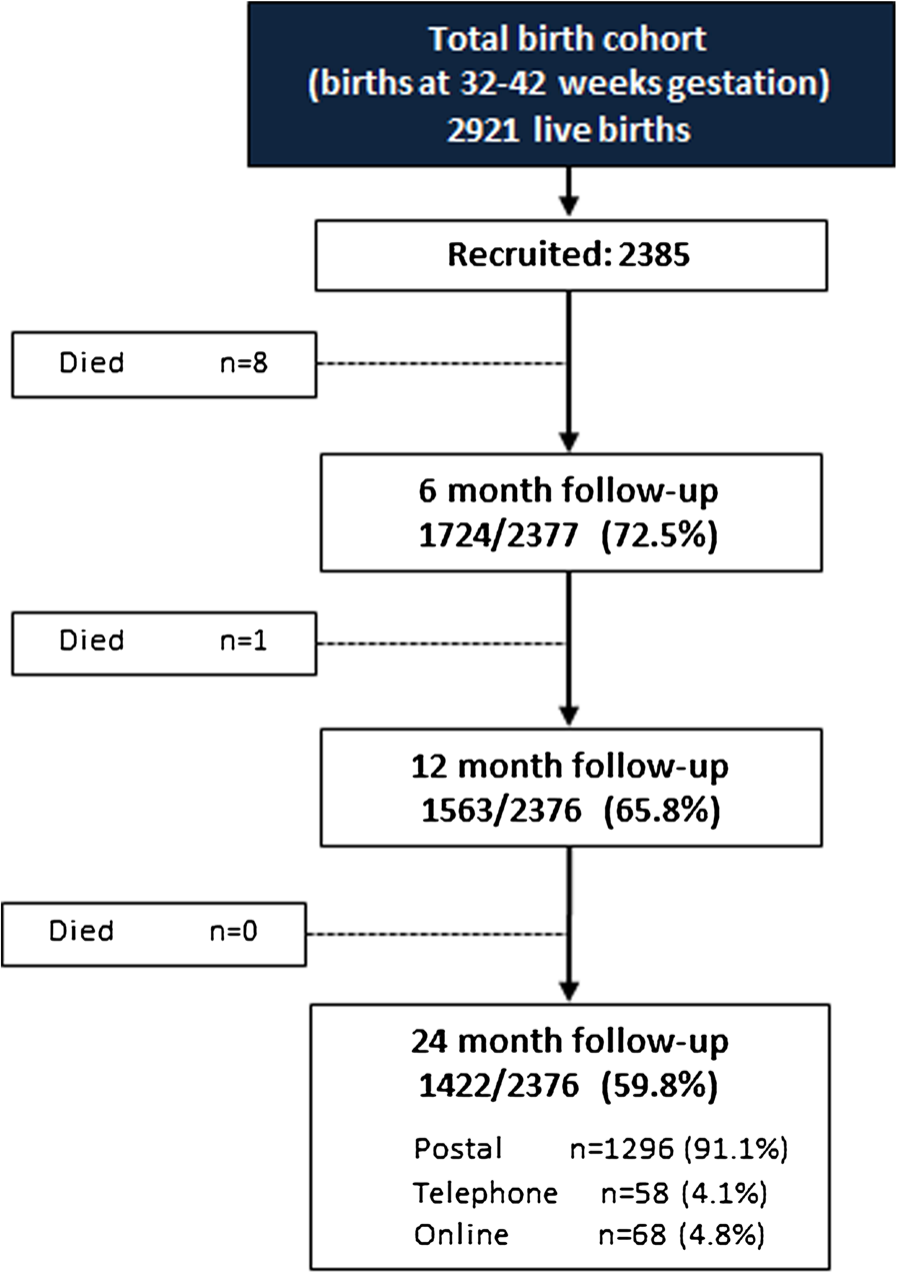
Study sample and response rates to 2 years corrected age (denominator = number of infants).

Of 2921 eligible live births, 2385 (82%) were recruited (Figure 2). Nine infants died following recruitment leaving 2376 children eligible for follow-up. At 2 years, the parents of 1422 children (59.8%) responded, of which 1296 (91%) completed a postal questionnaire, 58 (4%) a telephone interview and 68 (5%) an online questionnaire (Figure 2). Thus of the 1080 infants whose parents did not initially return a postal questionnaire, 11.7% ultimately provided data by telephone or online increasing the overall response rate by 5.3% from 54.5% to 59.8%.

2) *Do telephone interviews and online questionnaires improve sample representation?*

To answer this question, we compared the characteristics of non-responders with those who completed questionnaires by telephone or online. As shown in Table 1, non-responders had significantly greater socio-economic and demographic risk: these mothers were more likely to be younger, non-white, non-English speaking, less well educated, unemployed or in a manual or routine occupation, unsupported at home, finding it difficult to manage financially and to have poorer mental and general health (all p’s <0.05). They were also more likely to be mothers of multiples and less likely to be giving their child breast milk at discharge; however, no indices of poor neonatal outcome were significantly associated with non-response.

As shown in Table 2, mothers who completed questionnaires via telephone/online shared key demographic characteristics with non-responders: they were significantly more likely to be non-white, non-English speaking, have had a multiple birth and to have delivered at term (all p’s <0.05).

3) *Do telephone interviews and online questionnaires yield comparable rates of missing data?*

There were no missing data on the neurosensory items in either response mode (Table 3). In contrast, telephone/online completions resulted in significantly more missing data than postal questionnaires for the PARCA-R. However, this was due to online completions only; two parents abandoned the online questionnaire after the neurosensory items resulting in substantial portions of missing PARCA-R data for these children. When these two respondents were excluded, there was no significant difference in the proportion of missing PARCA-R data between response modes (Table 3).

4) *Do telephone interviews and online questionnaires yield comparable rates of adverse outcomes?*

The prevalence of adverse outcomes did not differ significantly between response modes (Table 3). Adjustment for factors that were significantly associated with response mode (i.e., ethnic group, language, multiple birth, preterm birth; see Table 2), and therefore potential confounders, did not change the conclusions (Table 3).

**Table 1:** Maternal and infant characteristics of responders and non-responders to 2-year neurodevelopmental follow-up

**Characteristic**	**Responders**	**Non-responders**	**RR [95% CI] for non-responder**	**p**
**Maternal characteristics**	**(n = 1292)**	**(n = 844)**		
Age, mean (SD)	30.6 (5.6)	28.0 (6.0)	0.95 [0.95 to 0.96]	<0.001
Non-white ethnic group, n (%)	250 (19.4)	279 (33.1)	1.50 [1.35 to 1.67]	<0.001
Non-English first language, n (%)	165 (13.0)	161 (19.5)	1.32 [1.16 to 1.50]	<0.001
Unsupported mother, n (%)	127 (9.8)	194 (23.0)	1.69 [1.51 to 1.88]	<0.001
Highest educational qualification, n (%)				
None or secondary education	415 (32.6)	448 (55.1)	Baseline	-
Tertiary education	760 (59.6)	296 (36.4)	0.54 [0.48 to 0.61]	<0.001
Other	99 (7.8)	69 (8.5)	0.80 [0.65 to 0.96]	0.02
Occupational status, n (%)^a^				
Managerial, professional or intermediate	742 (57.9)	269 (32.0)	Baseline	
Semi-routine or routine	184 (14.4)	137 (16.3)	1.60 [1.36 to 1.89]	<0.001
No occupation	355 (27.7)	436 (51.7)	2.07 [1.84 to 2.34]	<0.001
Difficult financial situation, n (%)	80 (6.2)	74 (8.8)	1.24 [1.04 to 1.47]	0.02
Poor mental health, n (%)	141 (11.0)	119 (14.1)	1.18 [1.02 to 1.37]	0.02
Poor general health, n (%)	83 (6.4)	87 (10.3)	1.33 [1.14 to 1.56]	<0.001
**Infant characteristics**	**(n = 1422)**	**(n = 954)**		
Late/moderately preterm, n (%)^b^	651 (45.8)	472 (49.5)	1.09 [0.99 to 1.21]	0.08
Birthweight, n (%)				
Low birthweight (1501-2500 g)	404 (28.4)	298 (31.2)	1.08 [0.98 to 1.20]	0.13
Very low birthweight (≤1500 g)	18 (1.3)	13 (1.4)	1.07 [0.70 to 1.63]	0.75
Fetal growth restriction, n (%)^c^	273 (19.2)	213 (22.3)	1.12 [1.00 to 1.25]	0.06
Multiple birth, n (%)	262 (18.4)	219 (23.0)	1.17 [1.05 to 1.31]	0.005
Any respiratory support, n (%)^d^	100 (7.0)	57 (6.0)	0.90 [0.73 to 1.11]	0.32
Intra-cranial abnormality, n (%)^e^	12 (0.8)	7 (0.7)	0.92 [0.51 to 1.66]	0.77
Major congenital anomaly, n (%)	16 (1.1)	13 (1.4)	1.12 [0.74 to 1.68]	0.60
Any breast milk at discharge, n (%)^f^	1001 (70.4)	519 (54.4)	0.67 [0.61 to 0.74]	<0.001

^a^Classified using UK National Statistics Socio-Economic Classification (NS-SEC). ^b^Late and moderately preterm is birth at 32-36 completed weeks of gestation. ^c^Fetal growth restriction calculated using customised antenatal growth charts [21]. ^d^Any respiratory support includes infants who were ventilated or received non-invasive respiratory support. ^e^Intra-cranial abnormality includes Grade III or IV intra-ventricular haemorrhage (IVH), periventricular leukomalacia (PVL) and Grade II or III neonatal encephalopathy. ^f^Includes breast milk fed by any method.

**Table 2:** Maternal and infant characteristics of responders via postal questionnaire vs. responders via telephone interview or online to 2-year neurodevelopmental follow-up

**Characteristic**	**Postal completion**	**Telephone or online completion**	**RR [95% CI] for telephone/online**	**p**
**Maternal characteristics**	**(n = 1183)**	**(n = 109)**		
Age at birth of study child, mean (SD)	30.6 (5.6)	30.8 (5.2)	1.01 [0.98 to 1.04]	0.68
Non-white ethnic group, n (%)	220 (18.6)	30 (27.8)	1.60 [1.08 to 2.38]	0.02
Non-English first language, n (%)	144 (12.4)	21 (19.6)	1.63 [1.04 to 2.56]	0.03
Unsupported mother, n (%)	119 (10.1)	8 (7.3)	0.73 [0.36 to 1.46]	0.37
Highest educational qualification, n (%)				
None or secondary education	385 (33.1)	30 (27.5)	Baseline	-
Tertiary education	690 (59.2)	70 (64.2)	1.27 [0.84 to 1.92]	0.25
Other	90 (7.7)	9 (8.3)	1.26 [0.62 to 2.56]	0.53
Occupational status, n (%)^a^				
Managerial, professional or intermediate	689 (58.6)	53 (50.0)	Baseline	-
Semi-routine or routine	164 (14.0)	20 (18.9)	1.52 [0.93 to 2.48]	0.09
No occupation	322 (27.4)	33 (31.1)	1.30 [0.86 to 1.97]	0.21
Difficult financial situation, n (%)	71 (6.0)	9 (8.3)	1.35 [0.71 to 2.58]	0.36
Poor mental health, n (%)	123 (10.4)	18 (16.7)	1.63 [1.01 to 2.61]	0.05
Poor general health, n (%)	73 (6.2)	10 (9.2)	1.47 [0.80 to 2.71]	0.22
**Infant characteristics**	**(n = 1296)**	**(n = 126)**		
Late/moderately preterm, n (%)^b^	607 (46.8)	44 (34.9)	0.64 [0.45, 0.90]	0.01
Birthweight, n (%)				
Low birthweight (1501-2500 g)	372 (28.7)	32 (25.4)	0.86 [0.59 to 1.27]	0.45
Very low birthweight (≤1500 g)	16 (1.2)	2 (1.6)	1.21 [0.32 to 4.53]	0.78
Fetal growth restriction, n (%)^c^	242 (18.7)	31 (24.6)	1.37 [0.94 to 2.02]	0.11
Multiple birth, n (%)	229 (17.7)	33 (26.2)	1.57 [1.08 to 2.28]	0.02
Any respiratory support, n (%)^d^	95 (7.3)	5 (4.0)	0.55 [0.23 to 1.31]	0.17
Intra-cranial abnormality, n (%)^e^	11 (0.9)	1 (0.8)	0.94 [0.14 to 6.18]	0.95
Major congenital anomaly, n (%)	16 (1.2)	0 (0.0)	-	-
Any breast milk at discharge, n (%)^f^	916 (70.7)	85 (67.5)	0.87 [0.61 to 1.24]	0.45

^a^Classified using UK National Statistics Socio-Economic Classification (NS-SEC). ^b^Late and moderately preterm is birth at 32-36 completed weeks of gestation. ^c^Fetal growth restriction calculated using customised antenatal growth charts [21]. ^d^Any respiratory support includes infants who were ventilated or received non-invasive respiratory support. ^e^Intra-cranial abnormality includes Grade III or IV intra-ventricular haemorrhage (IVH), periventricular leukomalacia (PVL) and Grade II or III neonatal encephalopathy. ^f^Includes breast milk fed by any method.

**Table 3:** Quality of data obtained on 2-year parent-reported neurodevelopmental outcome measures by mode of questionnaire completion

**Outcome measure**	**Postal (n = 1296)**	**Telephone/online (n = 126)**	**RR [95% CI]**	**p**
**Proportion of missing data, n (%)**				
Neurosensory impairment^b^	0 (0.0)	0 (0.0)	-	-
Cognitive impairment^a^	149 (0.3)	92 (2.1)	6.35 [1.97 to 20.47]	0.002
**Proportion of missing data excluding online premature terminations, n (%)**				
Neurosensory impairment	0 (0.0)	0 (0.0)	-	-
Cognitive impairment	149 (0.3)	24 (0.6)	1.68 [0.25 to 11.27]	0.59
**Prevalence of adverse outcomes, n (%)**				
Neurosensory impairment	30 (2.3)	5 (4.0)	1.71 [0.67 to 4.42]	0.26
Cognitive impairment	169 (13.3)	13 (10.8)	0.82 [0.46 to 1.44]	0.48
**Proportion of adverse outcomes adjusted for significant differences in response mode**^**c**^				
Neurosensory impairment	-	-	1.91 [0.73, 4.99]	0.19
Cognitive impairment	-	-	0.82 [0.46, 1.44]	0.40

Cognitive impairment was assessed using the Parent Report of Children’s Abilities-Revised (PARCA-R). ^a^The proportion of missing data was calculated using the 34 non-verbal sub-scale items alone. ^b^Neurosensory impairment was measured using 4 items assessing vision, hearing, communication and neuromotor function. ^c^Adjusted for ethnic group, language, multiple birth and preterm birth.

## Discussion

The results of this study show that offering telephone interviews and online questionnaires improves response rates and enhances sample representation over postal questionnaires alone, whilst retaining data completeness and unbiased outcomes. Although this has previously been shown in relation to adult self-report questionnaires, the utility of mixed mode data collection for assessing infant neurodevelopmental outcomes has not been explored.

Consistent with previous studies [2-4,6], mothers who did not respond to follow-up had lower socio-economic status, poorer health and were more likely to be from non-white ethnic groups. As these factors are associated with adverse neurodevelopmental outcomes, improving response rates is therefore important for reducing non-response bias [26]. Our results indicate that offering multiple modes of questionnaire completion is effective for enhancing both response rates and sample representation. Indeed, previous studies have shown that following up non-responders to postal surveys with telephone interviews or reminders improves response rates among participants from socio-economically deprived and ethnically diverse samples, consistent with the present results. Choosing to complete questionnaires via the telephone may have reflected mothers’ difficulty in completing postal questionnaires due to poor command of written English. Indeed, telephone interviews reduce the cognitive demands placed on participants compared with written questionnaires [13]. Given the poorer response rate to postal surveys among ethnic minority samples [27], these are pertinent considerations for studies in which ethnic minority groups may be over-represented.

In addition to improving response rates and sample representation, our results show that the completeness of data obtained via telephone interviews and online questionnaires was comparable with that of postal questionnaires. Moreover, there were no significant differences in the prevalence of adverse outcomes between response modes. This is a key finding for studies in which parent reports of neurodevelopment at 2 years constitute primary outcomes. The PARCA-R in particular is increasingly used as an outcome measure in RCTs of perinatal interventions, both nationally and internationally, and as a clinical follow-up tool [28-31]. As response mode did not bias outcomes, researchers can have added confidence in using multiple methods to collect data using this scale.

Telephone interviews and online questionnaires were associated with a greater proportion of missing data, but this does not negate the efficacy of these for data collection, especially in the case of telephone interviews. The excess of missing data arose as a result of two respondents who abandoned the online questionnaire prior to completing the PARCA-R. After excluding data from these respondents, there was no difference in the proportion of missing data, as shown previously in relation to telephone interviews [11,13,14]. It should be noted that we did not allow multiple completions of the online questionnaire from the same IP address; as parents had to complete the questionnaire on one occasion the premature terminations may be an artefact of this. Missing data may be minimised by allowing online questionnaires to be completed in multiple sittings. In addition, other practical issues should be considered when offering mixed mode data collection. Contacting parents by telephone to provide reminders and offer an interview was labour intensive and required substantial additional resources to those initially envisaged; multiple attempts were often needed to telephone parents before contact was made. The added value of offering these methods of follow-up should therefore be weighed against the resources available for maintaining contact with a cohort in this manner [32].

### Strengths and limitations

We believe our results will be applicable on a wider level. The challenges facing researchers are universal and the methods we explored are generic in nature. Our response rate was also comparable with that of postal surveys conducted in both healthcare and social science settings [27,33]. However, there are a number of limitations that may be addressed in future research. The 5% increase in response rates for offering multiple response modes was modest compared with previous studies [10,11]. However, these studies were conducted with adult self-report questionnaires and issues of non-response may be different in birth cohort studies. Our study was also limited by the use of a hierarchical design in which telephone and online response modes were offered to those who had not responded to a postal questionnaire in a timely manner, thus comprising a group who were already difficult to follow-up. It is impossible to determine how many parents would have opted for online or telephone completion had their preferred response mode been ascertained at study entry. Sending the initial request for follow-up using their preferred response mode may have substantially increased response rates, particularly for online questionnaires which are often preferred over postal questionnaires [13]. In addition, the relatively small number of non-postal responders meant that we were unable to assess differences between the three response modes separately or to explore these issues in relation to different ethnic or socio-economic groups. Future studies should therefore aim to replicate these results using large prospective studies in which participants are randomised to response modes. More studies of the validity of electronic data collection for neurodevelopmental follow-up are needed as such methods are likely to become a mainstay with ongoing advances in technology.

## Conclusions

Offering telephone interviews and online questionnaires as alternatives to postal questionnaires can enhance follow-up rates, improve sample representation and reduce non-response bias when assessing infant neurodevelopmental outcomes. Where the technology and resources allow, these measures may prove fruitful for maximising response rates and reducing non-response bias in birth cohort studies and RCTs of perinatal interventions. Larger studies are needed to replicate these findings using randomised allocation of response mode.

## Abbreviations

FGR: Fetal growth restriction; IVH: Intra-ventricular haemorrhage; NSI: Neurosensory impairment; PARCA-R: Parent Report of Children’s Abilities-Revised; PVL: Periventricular leukomalacia.

## Competing interests

The authors declare that they have no competing interests.

## Authors’ contributions

SJ designed and managed the 2-year neurodevelopmental outcome assessment and was responsible for interpreting study results and drafting and revising the manuscript. SES carried out statistical analyses and interpretation of study results and reviewed and revised the manuscript critically for intellectual content. BM, LKS, DF, ESD, NM & EMB conceived and designed the study, contributed to study management and reviewed the manuscript critically for intellectual content. DF was the principal investigator. All authors read and approved the final manuscript for submission.
